# Supplementary figures and images for: Persistently increased post-stress activity of paraventricular thalamic neurons is essential for the emergence of stress-induced alterations in behaviour
Source: PLoS Biol. 2025 Jan 21;23(1):e3002962. doi: 10.1371/journal.pbio.3002962 (PMC11750107; doi:10.1371/journal.pbio.3002962)

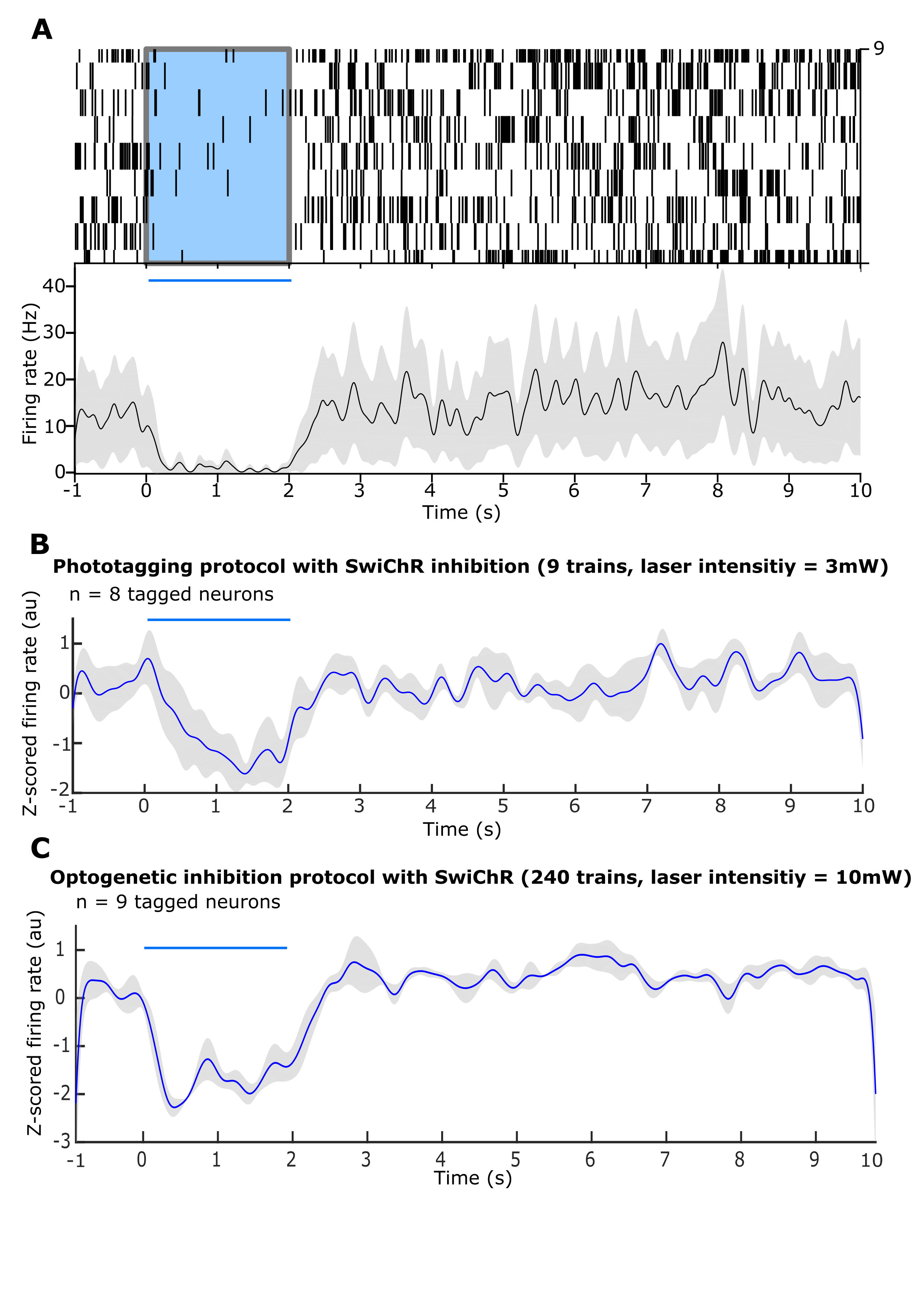

Supplement: S1 Fig — (A) Representative example of the firing rate of a PVT/CR+ neuron during 2 s laser ON 13 s laser OFF trains (n = 9 trains) in the wake state. Top Raster plot of one neuron during 9 trains. Blue rectangle marks the laser ON time (2 s). Bottom Firing rate for the same cell calculated in 10 ms windows. (B) Z-scored firing rate of 8 phototagged PVT/CR+ neurons using the 4 × 15 s phototagging protocol (see Methods). Mean (blue line) +/- SD (grey) are shown. Straight blue line above marks the laser ON time (2 s). (C) Z-scored firing rate of 9 phototagged PVT/CR+ neurons during the 60 min long photoinhibition protocol (see Methods). Mean (blue line) +/- SD (grey) are shown. Straight blue line above marks the laser ON time (2 s). (TIFF) [file pbio.3002962.s001.tiff]

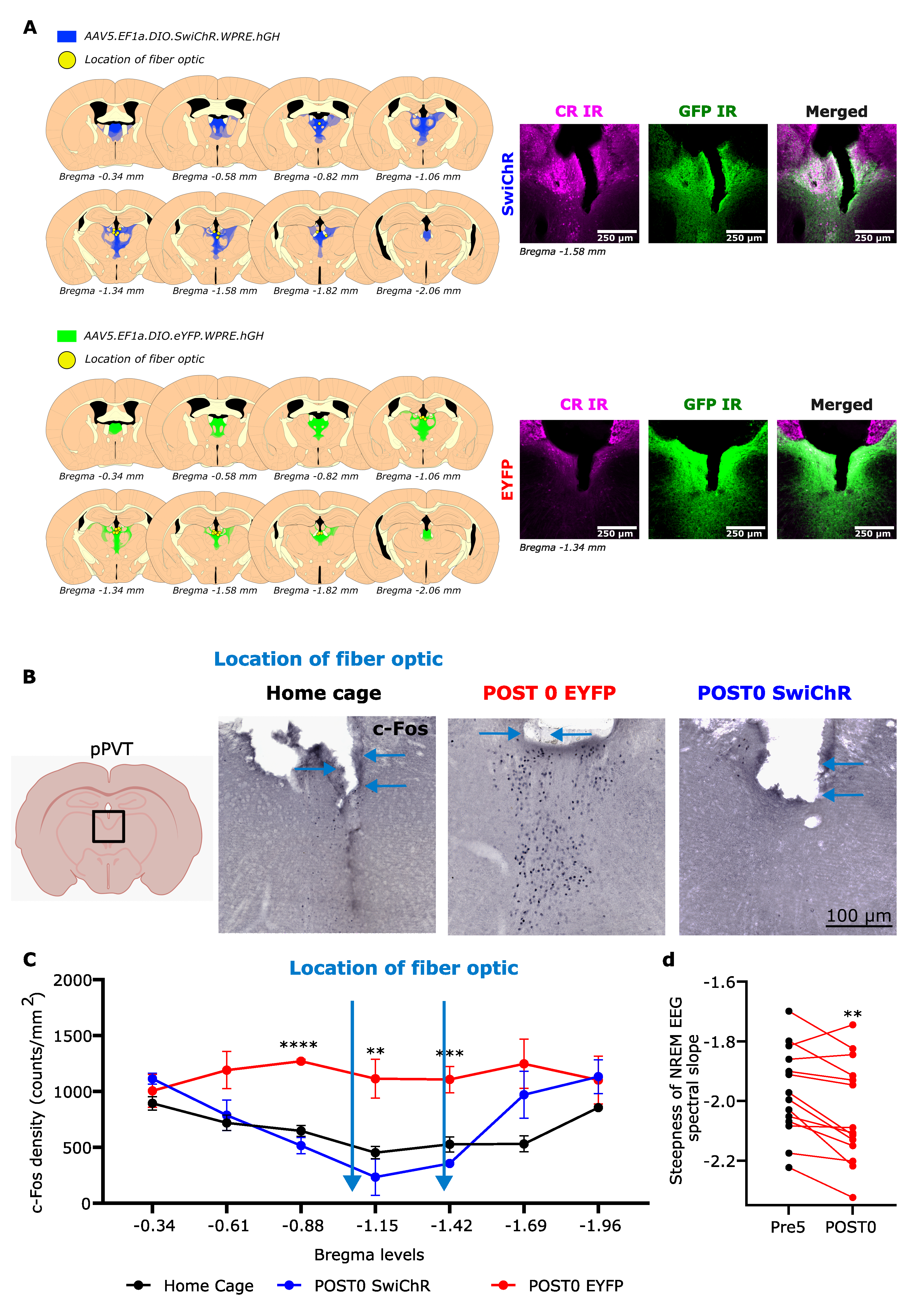

Supplement: S2 Fig — (A) Schematics of coronal sections illustrating the location of the optical fibres (yellow dots) and the extent of transfection following SwiChR (top, blue) and EYFP (bottom, green) virus constructs injected to the PVT of CR-Cre mice. Drawings are based on a compilation of 14 animals for EYFP and 7 for SwiChR. The schematics of coronal sections were created according to the Franklin and Paxinos mouse brain atlas [83]. Right, representative images depicting fibre optic tracks from SwiChR and EYFP injected CR-Cre mice. Green represent GFP immunolabelling, magenta represents CR immunolabelling. (B) Representative images showing c-Fos immunolabelling in the PVT with fibre optic tracks (blue arrows) from home cage control, EYFP, and SwiChR mice after POSE. Created with BioRender.com. (C) Quantification of c-Fos expression across the rostro-caudal extent of the PVT. Blue arrows mark the position of the fibre optics (−0.88 Bregma level, F(2,10) = 58.33, p = 0.0001; −1.15 Bregma level, F(2,13) = 12.44, p = 0.001; −1.42 Bregma level, F(2,11) = 18.53, p = 0.0003). (D) Comparison and the steepness of the NREM EEG spectral slope between the fifth day (black dots) of the pre-stress period and POST0 day (red dots) in EYFP mice (t[13] = 3.58, p = 0.0034). Dots represent individual animals. Underlying data can be found in S9 Data. See S10 Data for the full results of the statistical tests. Data are means ± SEM. *p < 0.05, **p < 0.01, ***p < 0.001, ****p < 0.0001. (TIFF) [file pbio.3002962.s002.tiff]

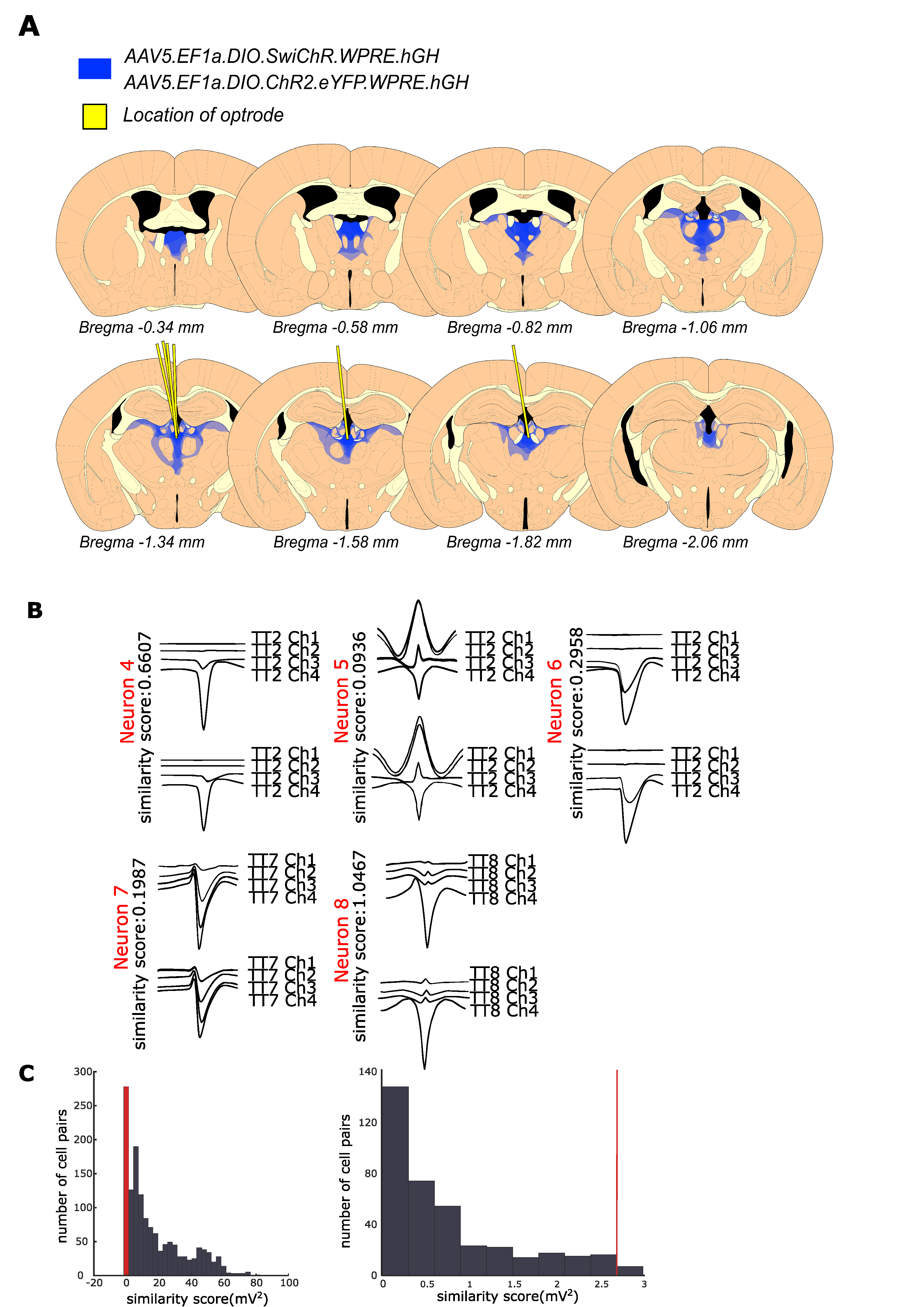

Supplement: S3 Fig — (A) Schematic of coronal sections illustrating the placement of tetrodes (yellow lines) and extent of injection sites using conditional ChR2 or SwiChR (blue) containing virus constructs targeted to the PVT in CR-Cre mice. Drawings are based on 6 mice (n = 4 for ChR2 and n = 2 for SwiChR). The schematic of coronal sections was created according to the Franklin and Paxinos mouse brain atlas [83]. (B) Representative waveforms of optotagged PVT/CR+ neurons in different tetrodes of the same animal shown in Fig 2E (tetrodes: TT2, TT7, TT8). The neurons were recorded for 2 consecutive days (top vs. bottom row). Channel numbers (Ch) and similarity scores (see Methods) between the 2 days are shown next to the waveforms. (C) Distribution of similarity scores of all tagged PVT/CR+ neurons from the ChR2 animals. Red line indicates the threshold. Left, all cell pairs. Right, zoomed in image showing the distribution of cell pair numbers within the range of the threshold. (TIFF) [file pbio.3002962.s003.tiff]

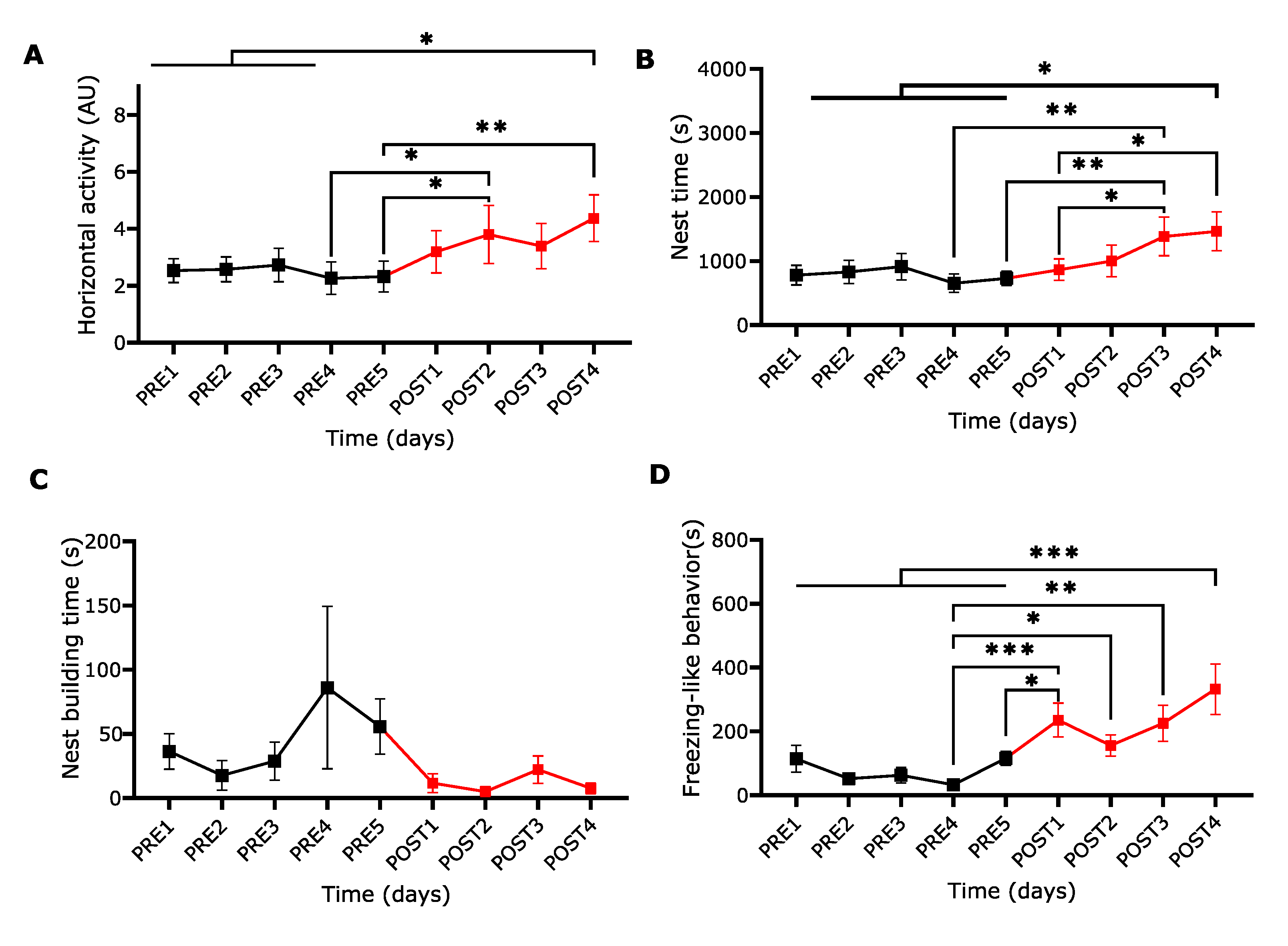

Supplement: S5 Fig — (A) Day by day normalised horizontal locomotor activity of EYFP animals during the PRE 1–5 and the POST 1–4 days (one-way RM-ANOVA followed by Fisher post hoc test, F(8,101) = 2.065, p < 0.05; PRE1 vs. POST4 p < 0.05; PRE2 vs. POST4 p < 0.05; PRE3 vs. POST4 p < 0.05; PRE4 vs. POST1 p < 0.05; PRE4 vs. POST3 p < 0.01; PRE5 vs. POST2 p < 0.05; PRE5 vs. POST4 p < 0.01). (B) Awake nest time duration of EYFP animals during the PRE 1–5 and the POST 1–4 days (one-way RM-ANOVA followed by Fisher post hoc test, F(8,98) = 2.542, p < 0.05; PRE1 vs. POST4 p < 0.05; PRE2 vs. POST4 p < 0.05; PRE3 vs. POST4 p < 0.05; PRE4 vs. POST3 p < 0.01; PRE4 vs. POST4 p < 0.01; PRE5 vs. POST3 p < 0.01; PRE5 vs. POST4 p < 0.01; POST1 vs. POST3 p < 0.05; POST1 vs. POST4 p < 0.05). (C) Time spent with nest building by EYFP animals during the PRE 1–5 and the POST 1–4 days (one-way RM-ANOVA F(8,99) = 1.272, p > 0.05). (D) Time spent with freezing-like behaviour by EYFP animals during the PRE 1–5 and the POST 1–4 days (one-way RM-ANOVA followed by Fisher post hoc test, F(8,100) = 5.774, p < 0.0001; PRE1 vs. POST4 p < 0.001; PRE2 vs. POST1 p < 0.01; PRE2 vs. POST3 p < 0.01; PRE2 vs. POST4 p < 0.0001; PRE3 vs. POST1 p < 0.01; PRE3 vs. POST3 p < 0.01; PRE3 vs. POST4 p < 0.0001; PRE4 vs. POST1 p < 0.001; PRE4 vs. POST2 p < 0.05; PRE4 vs. POST3 p < 0.01; PRE4 vs. POST4 p < 0.0001; PRE5 vs. POST1 p < 0.05; PRE5 vs. POST4 p < 0.001; POST2 vs. POST4 p < 0.01). (TIFF) [file pbio.3002962.s005.tiff]

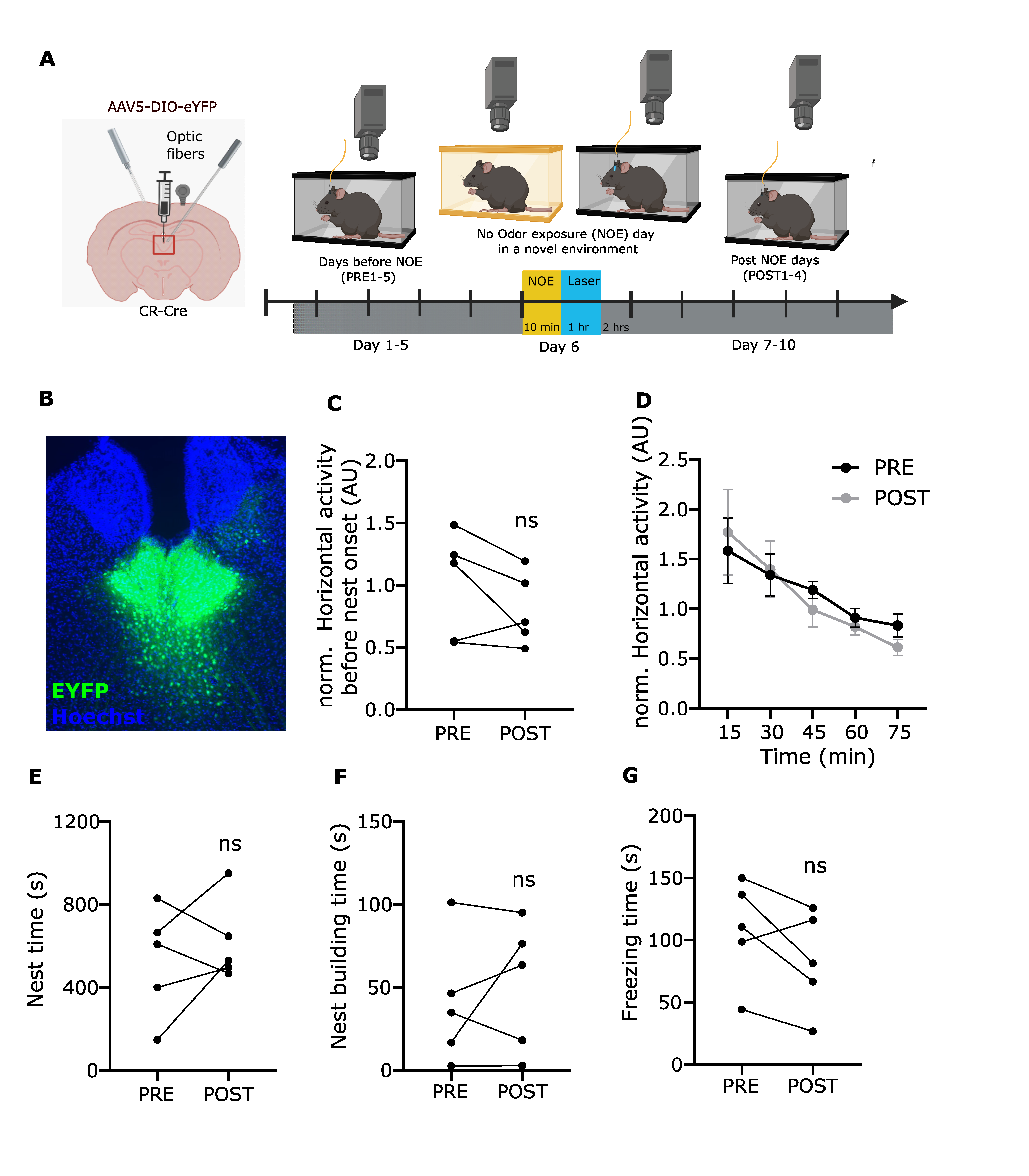

Supplement: S6 Fig — (A) Scheme of the experiment. EYFP-injected CR-Cre mice were exposed to a novel environment without predator odour to assess the effects of environmental novelty on home cage behaviour before (PRE) and after novelty exposure (POST). Created with BioRender.com. (B) Schematics of coronal section depicting the extent of transfection following EYFP (green) virus constructs injected to the PVT of CR-Cre mice. (C) The averaged, normalised horizontal locomotor activity of EYFP-injected CR-Cre mice exposed before nest onset (t[4] = 1.642, p = 0.1759). Dots represent the averaged daily values of individual animals. (D) Temporal dynamics of averaged, normalised horizontal locomotor activity of EYFP the animals (n = 5) during the PRE1–5 days (black) vs. the POST1–4 days (grey) periods. (E–G) (E) Nest time (t[4] = 0.7887, p = 0.4744), (F) nest building time (t[4] = 0.8094, p = 0.4637), (G) freezing time (t[4] = 1.965, p = 0.1208) in EYFP mice (n = 5) during the PRE and POST period. Dots represent the averaged daily values of individual animals. Underlying data can be found in S9 Data. See S10 Data for the full results of the statistical tests. Data are shown as mean ± SEM. (TIFF) [file pbio.3002962.s006.tiff]

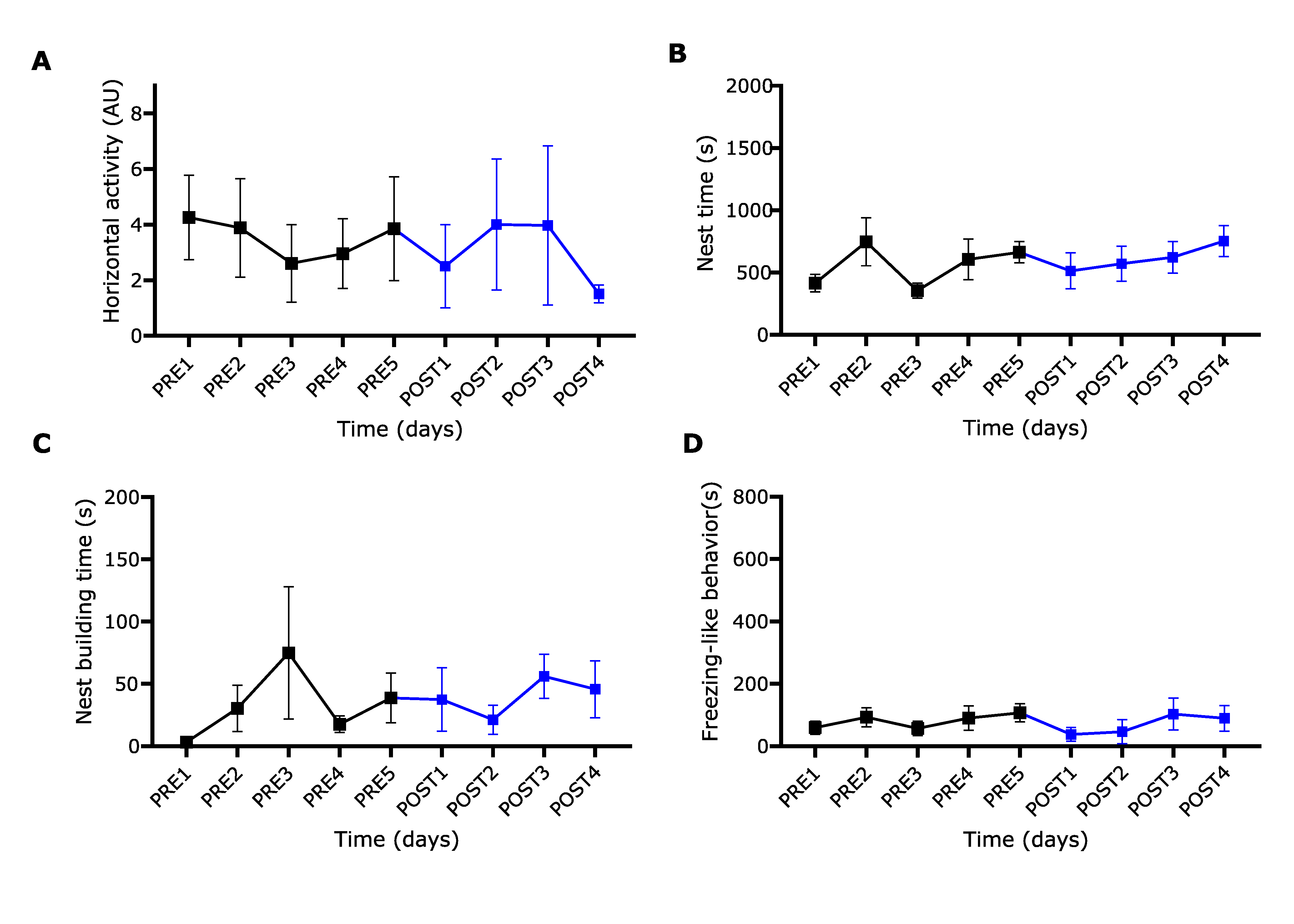

Supplement: S8 Fig — (A) Day by day normalised horizontal locomotor activity of SwiChR animals during the PRE 1–5 and the POST 1–4 days (one-way RM-ANOVA followed by Fisher post hoc test, F(8,44) = 1.129, p = 0.363). (B) Awake nest time duration of SwiChR animals during the PRE 1–5 and the POST 1–4 days (one-way RM-ANOVA followed by Fisher post hoc test, F(8,45) = 1.161, p = 0.344). (C) Time spent with nest building by SwiChR animals during the PRE 1–5 and the POST 1–4 days (one-way RM-ANOVA F(8,45) = 0.816, p = 0.596). (D) Time spent with freezing-like behaviour by SwiChR animals during the PRE 1–5 and the POST 1–4 days (one-way RM-ANOVA followed by Fisher post hoc test, F(8,45) = 0.6272, p = 0.7505). (TIFF) [file pbio.3002962.s008.tiff]

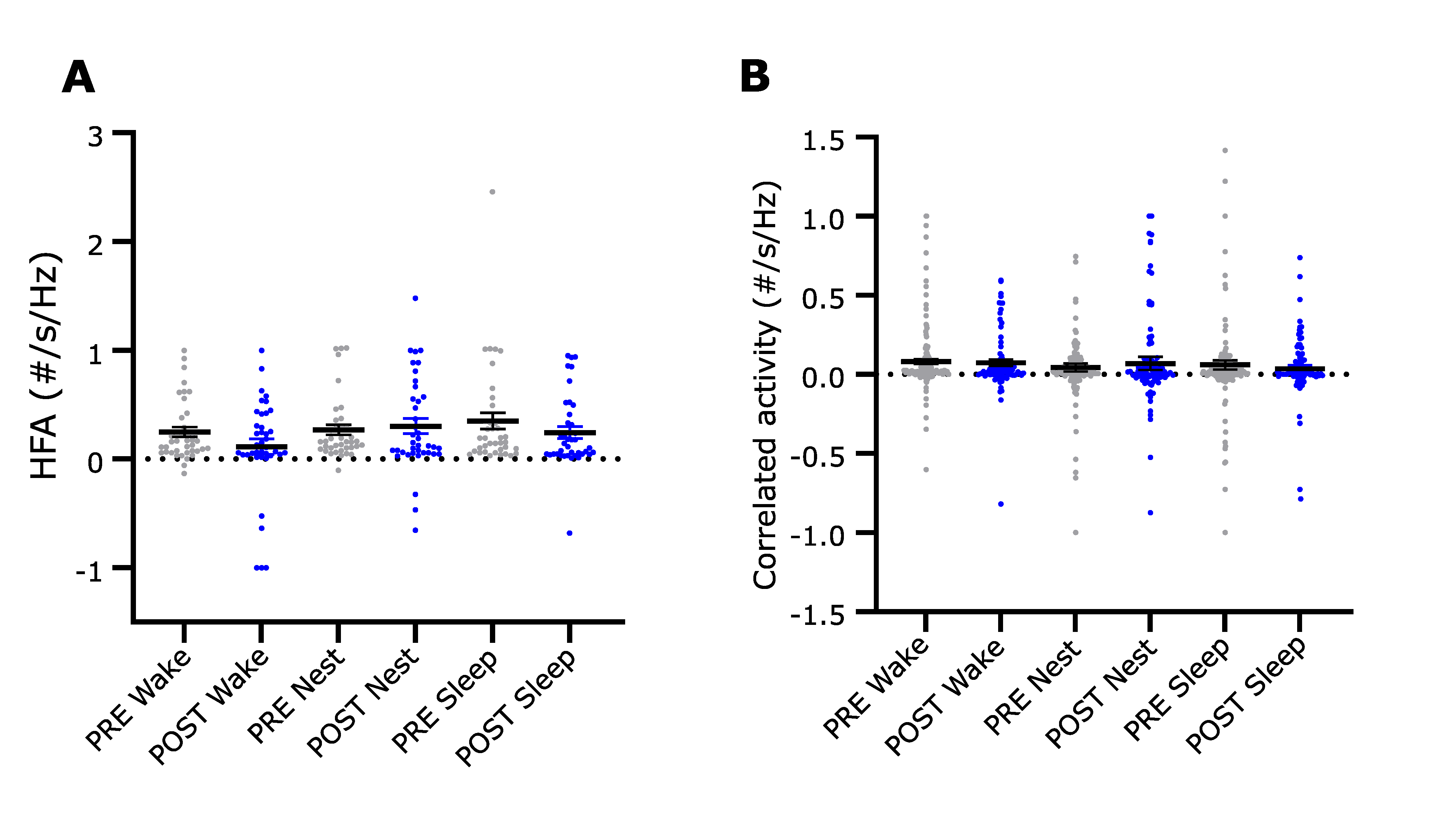

Supplement: S10 Fig — (A) Normalised HFA values with firing rate (see Methods) of the recorded and tagged neurons from inhibited (SwiChR) animals on the PRE (n = 38 from 2 animals) and the POST (n = 39 neurons from 2 animals) period (wake, U = 870.5, p = 0.5301; nest, U = 891, p = 0.6496; sleep U = 862, p = 0.4854). (B) Normalised CA values with baseline activity (see Methods) of the recorded and tagged neurons from control (SwiChR) animals on the PRE (n = 38 from 2 animals) and the POST (n = 39 neurons from 2 animals) period (U = 7,306, p = 0.552; nest, U = 7,418, p = 0.6884; sleep U = 7,356, p = 0.6106). Underlying data can be found in S9 Data. See S10 Data for the full results of the statistical tests. Data are means ± SEM. (TIFF) [file pbio.3002962.s010.tiff]
