# Supplementary material for: Persistently increased post-stress activity of paraventricular thalamic neurons is essential for the emergence of stress-induced alterations in behaviour
Source: PLoS Biol. 2025 Jan 21;23(1):e3002962. doi: 10.1371/journal.pbio.3002962 (PMC11750107; doi:10.1371/journal.pbio.3002962)
Supplement: Attachment — Submitted filename: Reviewers_Response_1009_final.docx [file pbio.3002962.s030.docx]

**RESPONSE TO REVIEWERS**

Reviewer #1: This study investigates the initial steps leading to enduring changes in behavior caused by acute stress exposure. The authors show that a single episode stressor increased the firing activity of calretinin-positive neurons within the PVT, which persisted for several days. When the authors inhibited these neurons for 1 hour after the stress exposure, it attenuated the increase in firing rate as well as the stress-induced behavioral changes. This manipulation also reduced activation of downstream targets associated with defensive behaviors, BLA, prelimbic and nucleus accumbens. Surprisingly, photoinhibiting the same neurons 5 days after the stress produced similar attenuations. The data are convincing that PVT/CR+ neurons are persistently changed after stress leading to altered behaviors. I do have some questions, mostly related to the analyses and interpretations.

1. In Fig 2e, the authors show 3 sample neurons across 2 sessions, pre and post stress, with a calculated "similarity score" for each neuron. How was this score calculated? What does a score 0.3 really mean? In the methods (LINE 1080), the authors report the similarity score ranged from "0 and 1.32". Presumably 0 means not similar. Why were these neurons included in the pre/post stress analyses?

Should there not be a cutoff criterion for a neuron's similarity score to be included in the analyses? In Suppl 3c, I am not understanding the y-axis of just "histogram" and the x-axis says "similarity score" but shows a range of 0 to 100, when actual similarity scores were 0-1.32. What does the red threshold marker mean? Threshold to calculate a similarity score? Threshold to be included in the analyses?

We apologise for the inaccuracies in Figure Supplementary 3C. We calculated the similarity score by taking the sum of the squared timepoint-by-timepoint differences in the amplitude of the waveforms in all the 4 channels (Supplementary Fig. 3b) among every unit recorded in the same tetrodes of an animal in any day (PRE1 to POST4 days). These values will be high when waveforms differ a lot thus the recorded signals belong to different cells. On the other hand, the similarity score will be low in those cases when we recorded the same unit on the same electrodes on two consecutive days thus there will be little difference between the waveforms.

In our case the lowest value of the similarity score was 0.0148 µV^2^ and the highest value was 80.3909 µV^2^. The cutoff value was the lowest 5% of the similarity scores. In our case this was 2.75 µV^2^, as a consequence, 0.3 µV^2^ is well below this value. In Fig 2E waveform similarity is also visually obvious in this case. We labelled the cutoff value with red in Supplementary Figure 3C. In order to better resolve this range, we prepared now a zoomed-in version of the same graph and show it in the new Supplementary Figure 3D. We altogether had 1404 pairs in the four animals, of these 334 pair met our criteria. From these pairs, in the present manuscript we used only those units which we could record before the stress (PRE 5 day) and immediately after the stress (POST0 days) since we were interested in how single PVT/CR+ units responded to the stress challenge (20 units with similarity score ranges between 0.0148 µV^2^ and 1.3198µV^2^). We now provide a more accurate description of the similarity score on page 57, lines 1108-1113.

2. For phototagging, what was the criterion for a phototagged cell? Within how many ms did the neuron need to respond to be considered phototagged? Is it possible that CR- neurons that received input from CR+ cells were included as phototagged CR+ cells? The x-axis in Suppl 4d is also not labeled.

We used Stimulus-Associated spike Latency Test (SALT), to identify significantly light-activated PVT/CR+ neurons in ChR2 animals (Kvitsiani et al., 2013). This test determines whether light pulses significantly changed the spike timing of a neuron by comparing the distribution of first spike latencies relative to the light pulse, assessed in a 10-msec window after light-stimulation, to 10-msec epochs in the baseline period (-150 to 0 msec from the onset of light-stimulation; (Kvitsiani et al., 2013) for details). A significance level of p < 0.01 were considered identified CR+ neurons. For clarity, we have included this description in the Methods section on page 58, lines 1115-1119.

According to our current knowledge, there is no monosynaptic connection among thalamocortical cells. Individual cell labelling has never consistently resolved recurrent collaterals emitted by thalamocortical cells within the thalamus. Similarly, no cell pair with monosynaptic connection was found using physiological methods. In sensory nuclei a low percentage of the neurons (15-20%) are coupled via gap-junctions, however, the connectional strength is not sufficient to allow action potential coupling. As a consequence, inclusion of misidentified, synaptically coupled CR- cells can be excluded beyond reasonable doubts.

3. For the experiment where photoinhibition was applied 5 days after the stress leading to reversal of many of the behaviors, etc is it not surprising that a single photoinhibition session so many days after the stressor could have such long-lasting effects? The first photoinhibition experiment, where it was applied right after the POSE, makes more sense because it is presumably interfering with the consolidation/induction of the hyperactivity. However, once the hyperactivity was established how does only 1 photoinhibition session seemingly reverse everything? There is next to nothing about this in the discussion.

Thank you for your insightful question regarding the effects of photoinhibition applied 5 days post-stress and the apparent long-lasting impact on behavior. We appreciate the opportunity to clarify this aspect of our study.

The results indicating that a single session of photoinhibition 5 days after the stressor could reverse many of the stress-induced behavioral alterations are indeed surprising and completely unexpected. We think, however, that this effect is in line with our other observations from our study on the peculiar, long-term changes of neuronal activity in PVT/CR+ cells in response to environmental challenges. Our data clearly demonstrate that PVT/CR+ neurons are endowed with mechanisms that allow them to alter their firing rate for a prolonged time in response to a transient impact, be it behavioral or optogenetic. We must emphasize that we regard this type of “plasticity” distinct from all those described before, and its exact mechanisms need to be clarified in the future.

In our earlier study (Mátyás et al., 2018) we found that PVT/CR+ cells have highly diverse outputs to the key forebrain centers involved in stress and they can exert strong impact on their activity. As a consequence, altered PVT/CR+ neuronal activity will always be reflected in a corresponding change in the activity of their targets. Our c-Fos data (Figure 7) in the present manuscript clearly confirms this prediction. Altered forebrain activity in turn will be responsible for the altered behavior.

The key point is that PVT/CR+ activity can not only change for prolonged periods in the positive direction but transient inhibition via optogenetics can also result in a long-term decrease of PVT/CR+ activity and, consequently, the associated stress-related behaviors. Our data show that in the EYFP group PVT/CR+ activity is still high 5 days after the stress. This stress induced elevation can be blocked by photoinhibition at day 0 for the entire 5 days period. Our interpretation is that the hyperpolarization (Cl^-^ influx) we induced by means of optogenetics after the stress reversed the mechanisms that are responsible for the prolonged change in firing rate. Note here that, had the increased firing rate been caused by elevated activity of the afferents, PVT/CR+ activity would immediately return to the elevated level after the cessation of photoinhibition. But this clearly did happen. Rather the data show that photoinhibition at day 0 can reverse PVT/CR+ activity for a prolonged period five days after the stress exposure.

We can assume that we will have the same effect on behavior as long as the stress-induced behavior depends on the elevated PVT activity. Once the altered activity is translocated (i.e. consolidated) to the forebrain targets PVT inhibition will certainly no longer have any effect on the behavior. It must be kept in mind that the observed reversal in stress induced behavior does not imply complete normalization but rather a significant reduction in the stress-induced behavioral symptoms.

The novel experiments we performed in response to Review 2 point C1 (New Supplementary Figure 9) further confirm this view. In these experiments we inhibited animals that were exposed to the novel environment without the fox odor. In control conditions, this challenge leads to no significant change in the animals’ behavior. However, if we inhibit PVT/CR+ cells on Day 0, after this “no odor” condition stress-related behaviors improved compared to the control period. Animals become “calmer” than normal. This indicates again that a single interference with PVT neuronal activity in the negative direction can have prolonged effect on behavior. Collectively we regard the PVT/CR+ neurons as a “tunable device” that can maintain the impact of events that inducing both elevated and decreased activity for prolonged periods and induce change in stress-related behaviors accordingly.

We acknowledge that our initial discussion did not fully address the implications of applying photoinhibition 5 days post-stress. We updated the discussion to include an exploration of the potential mechanisms underlying the observed long-lasting effects of late photoinhibition. This involved a consideration of how the ongoing influence of elevated PVT/CR+ activity and associated plasticity could be disrupted by a single intervention, thereby reversing stress-induced behavioral changes. For a more succinct discussion of this point, see page 20, lines 473-482.

4. In Fig 8b,c, what is the data normalized to? Fig 8c shows LID has reduced horizontal activity and yet Fig 8e shows reduced freezing compared to POST. These don't seem to make sense together. How can there be reduced movement and reduced freezing? What is being analyzed differently between these 2 metrics?

In every case when normalized data are shown the normalization follows a consistent method. We normalize all behaviors and firing rates to the average pre-stress values of the animals. The apparent contradiction of the data - how both freezing and horizontal movement can be reduced after photoinhibition - can be explained by the fact that these two behaviors consist of only a fraction of the total time. In addition, the two behaviors are recorded at different times: horizontal activity is measured before the animals enter the nest, while freezing is measured once they are inside the nest. Stress is known to induce both hypervigilance (measured here as increased horizontal activity) and negative emotional change (measured here as freezing in the nest, which is supposed to be a safe place). In our behavioral paradigm, both types of behaviors are affected indicating that the behavior is altered inside as well as outside the nest. Regarding Figure 8, we updated the figure legend for details related to the normalization.

5. The authors mention in the introduction that the PVT/CR+ neurons have diurnal alterations in activity. Since the authors had long term recordings, including wake/sleep data, did the authors observe disruptions to the diurnal rhythm of these neurons' activity? Given the other data presented, it seems that this would happen. Related, did the sleep stages change after stress (i.e. non-REM vs REM).

Thank you for this question. Although our study observed long-term changes in PVT/CR+ neuron activity and behavior following stress, we did not specifically analyze the disruptions of diurnal rhythms. This was due to our experimental design, which focused on long-term recordings but did not include continuous 24-hour monitoring. As described in the Method section we recorded the first 3 hours after the onset of the light period for every mice. Our data primarily aimed to capture stress-induced alterations in neuronal firing rates and subsequent behavioral changes, rather than detailed diurnal patterns.

Regarding sleep stages, we measured sleep depth using a sophisticated method developed to measure human sleep depth (Figure 3J and Supplementary Figure 2D) and recorded changes in pre-sleep behaviors such as increased latency to fall asleep. However, we did not analyze changes in sleep stages (e.g., non-REM vs. REM) in detail. A major reason for this decision is that in our dataset we found many transient states between REM and nREM sleeps. Accurately distinguishing sleep stages requires extensive and continuous recordings, and the challenges of unequivocally assigning sleep stages made this a less feasible focus for our study. Future research with continuous 24-hour recordings and advanced techniques for sleep stage analysis could provide deeper insights into the impact of stress on diurnal rhythms and sleep patterns.

6. A prior study (Hua et al 2018, Current Biology) also looked at PVT/CR+ neurons in sleep/wake disturbances after starvation (a type of chronic stress) and showed the PVT-BNST pathway to be important. Can the authors comment on why BNST was not included in the present study?

Thank you for the insightful suggestion regarding the inclusion of the BNST in our study. Following your recommendation, we performed a thorough analysis of the BNST, specifically focusing on the oval BNST, as this region is known to receive input from the PVT. However, our One-Way ANOVA results did not reveal a significant effect of the stressor on c-Fos expression in the BNST (F(3, 20) = 0.8483, p = 0.4837). This indicates that the acute stressor used in our study (2MT exposure) did not significantly alter c-Fos expression in the BNST compared to home cage controls or the SwiChR (PVT-inhibited) and novel environment control groups.

This finding contrasts with the results of (Hua et al., 2018), which examined the PVT-BNST pathway under chronic stress induced by starvation. The discrepancy may be attributed to the different nature of chronic stress and metabolic stressors compared to our acute stressor might explain the discrepancy. Additionally, while the dorsolateral or oval BNST receives input from the PVT, the intra-BNST connectivity among its various nuclei is highly intricate and was not the primary focus of our study. We appreciate your attention to this aspect and the opportunity to clarify our findings. The new findings are shown in Fig. 7G, on pages 13-14, lines 312-320.

7. The authors do a nice job describing CR+ neurons of the PVT and their distribution and function. It might also be worth adding to the intro and/or discussion an overview of calretinin itself and its function and how it is able to support persistently higher neural activity.

Thank you for the suggestion to elaborate on calretinin’s (CR) and its role in supporting persistent neural activity. We appreciate your feedback and agree that a discussion of CR's functional significance would enhance our manuscript.

Calretinin is a calcium-binding protein that is widely expressed in various neuronal populations, including the PVT/CR+ neurons we investigated. It plays a critical role in regulating intracellular calcium levels, which in turn influences neuronal excitability and signaling. CR’s capacity to buffer intracellular calcium helps modulate neuronal firing rates and can support sustained changes in neural activity. This buffering capacity is crucial for maintaining stability in neural circuits during prolonged periods of heightened activity.

The involvement of calretinin in supporting a persistently high neural activity is particularly relevant in our study. In PVT/CR+ neurons, CR helps to stabilize excitatory signaling by managing calcium fluctuations that occur during stress-induced hyperactivity. This mechanism allows these neurons to maintain elevated firing rates over extended periods, contributing to the observed long-term changes in behavior and neural activity post-stress. Based on this suggestion, we mention the putative role of CR in supporting the maintenance of increased firing activity on page 4, lines 80-82.

8. LINE 382-3: Something is weird about the grammar of this particular sentence.
The sentence has been rewritten.

9. Figs 2, 3, and 4 have a schematic showing laser photoinhibition for 1h after POSE, but it does not appear that any of the data in these figures are from photoinhibited animals?

The animals shown in Figures 2, 3, and 4 expressed control opsin that contains only EYFP but not the inhibitory opsin SwiChR. To assure identical treatment of the EYFP and SwiChR group we applied the same 1-hour laser light stimulation using the same regime (2 sec ON 13 s OFF). Thus, in the EYFP group the PVT/CR+ neurons were not optically inhibited and, consequently, these animals developed stress induced alteration in behavior. The schematic was intended to show the experimental design and timing, but the data in these figures do not reflect the effects of photoinhibition. We clarified this distinction in the legend of the revised figures and accompanying text to ensure accurate representation of the experimental conditions. The blue light application in the EYFP-expressing animals was included as a technical parameter to provide a more accurate control for the SwiChR-mediated PVT/CR+ inhibition experiments. By applying the same 1-hour light stimulation regime (2 sec ON, 13 sec OFF) across both groups, we ensured that any observed differences in subsequent experiments could be attributed specifically to the presence of the SwiChR opsin, rather than to non-specific effects of the light itself. This setup strengthens the rigor and comparability of the findings presented in the subsequent figures.

10. I could not find "defensive behaviors" defined for Fig 1d.

Thank you for your question regarding the definition of "defensive behaviors" in the context of Fig 1d. In our study, defensive behaviors were operationally defined that included both escape jumps, and freezing responses observed during the 10-minutes exposure to 2MT. Notably, freezing accounted for 95% of the total time spent on defensive behaviors. These behaviors are commonly used in stress and fear research to gauge an animal's defensive reaction to a perceived threat.

To support this, we refer to Blanchard and Blanchard's work (Blanchard et al., 2001), which provides a comprehensive discussion on defensive behaviors such as freezing and escape responses. Their research explores how these behaviors are indicative of fear and anxiety in animal models. This foundational study outlines the use of these behaviors as key measures of defensive responses, which aligns with the observations reported in Fig 1d of our manuscript. We included the freezing time on page 6, line 128.

**Reviewer #2:**
This paper explores the role of the paraventricular nucleus of the thalamus (PVT) in the long-term effects of traumatic events, employing a combination of predator odor stimulation and long-term neural activity recordings via tetrodes. While the role of the PVT in stressed behaviors has been extensively studied, previous research has primarily focused on event-locked activities, with few analyzing longitudinal activity. Although I cannot confirm if this paper is indeed the first to report long-term neural activation, the authors present a unique approach and successfully elucidate PVT activities in enduring stressed behaviors.

Major points:
A) Virus reporter specificity: The authors should address concerns regarding the specificity of the virus reporters, particularly Calretinin/Calb2-Cre, which are not exclusively expressed in the PVT but also in neighboring thalamic nuclei. Supplementary Figures 3, 10, and 11 indicate significant non-PVT expression of Cre reporters. The authors should carefully analyze the "off-target" expression and its implications, including the positions of infected somas and the expression of c-fos and GABA-R in non-PVT CR+ nuclei. Discussion on the potential effects of off-target expressions is warranted, as strong off-target effects could shift the study's focus to the midline thalamus rather than the PVT.

Thank you for this question, this allows us to clarify in detail our steps to avoid “off-target” effects.

First, we carefully plotted the extent of virus in all our animals in Supplementary Figure 2, 3, 11 and 12 throughout the entire rostro-caudal extent of PVT and the surrounding nuclei. Indeed, substantial spread of the virus has been observed in the most dorsal aspect of the laterodorsal (LD) and lataroposterior (LP) nuclei close to the ventricle as well as in the rostral intralaminar nuclei including the centrolateral, paracentral and centromedian nuclei since all these nuclei are known to express calretinin.

In case of Supplementary Figure 3 the overspill of the virus did not affect our results since these experiments included tetrode recordings and the virus expression (ChR2 or SwiChR) is only used for tagging. Since the tertrode track is clearly inside the PVT in all cases, expression of the virus in the other nuclei did interfere with optogenetic identification of PVT/CR+ neurons.

In case of the Supplementary Figure 11 and Figure 7 we measured the GABA-A receptor expression in the EYFP control and SwiChR injected animals. In these cases, we carefully selected ROIs to measure the change in the level of GABA-A receptors close to the tip of the fiber optic which were always in PVT thus “off-target” expression is not expected to interfere with these data.

“Off-target” expression is the most critical in SwiCh2 experiments shown in Supplementary Figures 2, 9, 12 involving behavioral measurements. In these cases, inhibition of nuclei other than PVT/CR+ could indeed potentially interfere with the interpretation of our data. In order to circumvent this problem, we took a radical step, generally not used in the published photoinhibition experiments and measured the spatial extent of the photoinhibition by c-Fos expression.

Both this and our previous study (Mátyás et al., 2018) clearly indicated that stress induces c-Fos expression specifically in PVT/CR+ cells. We found that the SwiChR inhibition, as used here, is able to block this elevation of c-Fos expression (Figure 1J,K; Supplementary Figure 2C). As a consequence, drop in c-Fos expression is a very good proxy for the spatial spread of the photoinhibition. Thus, in order to validate the effectiveness and specificity of our inhibition, we measured the spatial extent of c-Fos expression relative to the fiber optics (Supplementary Figure 2C). We found that the effectiveness of photoinhibition does not spread further than 500 µm from the tip of the fiber optic. This shows with the laser power used in this study, that it is certainly not sufficient to reach the intralaminar nuclei, let alone the laterodorsal nucleus.

In order to directly demonstrate the selectivity of our approach here we provide an image, for reviewing purposes only, that demonstrates elevated c-Fos level in the intralaminar CR+ cells in the SwiChR animals after the photoinhibition of PVT.

**Fig. 1 for reviewing purposes only.** Lack of 'off-target' effects in the SwiChR experiments. A) Experimental design. B) Low-power light micrograph showing SwiChR-EYFP and c-Fos expression in the medial thalamus after POSE and SwiChR inhibition. C) Reduction of c-Fos expression is limited to the PVT and does not extend to the laterodorsal nucleus (LP) or the centromedial nucleus (CM) of the intralaminar complex (IL).

In sum, the positioning of the fiber optics in our optogenetic experiments was carefully controlled to ensure that light delivery was targeted specifically to the PVT, minimizing the potential for activation or inhibition of neighboring regions such as the laterodorsal or intralaminar nuclei. Given the precise placement of the fibers, it is unlikely that significant illumination occurred in these adjacent areas. In light of these considerations, we believe that the off-target expression of the Cre reporter and any associated effects on adjacent thalamic regions are unlikely to have shifted the focus of our study away from the PVT. Nonetheless, we included a discussion of these points on page 19, lines 445-447 in the revised manuscript to address the potential implications of off-target effects, ensuring that the interpretation of our results remains accurate and focused on the PVT. We appreciate the reviewer's suggestion and will ensure these details are clearly communicated in our discussion.

B) Regional Functionality: While previous reports highlight functional and characteristic distinctions between anterior and posterior PVT, the current manuscript lacks mention of regional specificity and utilizes manipulation between aPVT and pPVT. The authors should discuss regional specificity of PVT and justify their approach. Elaborating on Supplemental Figure 2C could strengthen their claim by revealing which part of PVT (and other CR+ nuclei) is activated by POSE and inhibited by SwitChR.

Thank you for your suggestion regarding the regional specificity within the PVT, particularly the distinction between anterior (aPVT) and posterior PVT (pPVT). We appreciate the opportunity to clarify and justify our approach.

In our study, we provided detailed coordinates for all manipulations, ensuring transparency regarding the regions of the PVT that were targeted without specific reference for aPVT or pPVT due to the following reasons. The exact border between the aPVT and pPVT is heavily contested and the border is drawn at different AP coordinates without an exact definition of the border. Presently, we have an ongoing project dedicated to draw a reliable border between aPVT and pPVT using connectivity as well as long term neuronal recording via multishank silicone probes implanted parasagitally in a way that it spans the aPVT-pPVT border. These data show that a morphologically and functionally distinct aPVT can be defined at the very rostral end of the nucleus by its connectivity with the hippocampal formation, more specifically with the ventral subiculum. In this aPVT sector, as it might be expected, PVT neuronal firing follows the hippocampal activity (theta, sharp wave) which is not present in the rest of the nucleus. This subicular input has already been described (Canteras & Swanson, 1992), but has not been used as a marker to differentiate aPVT and pPVT.

The aPVT-pPVT border as defined in this manner is much more rostral than what has been used in the literature. Thus, we intentionally avoided using the term “pPVT” in our study since it would have meant a more caudal region than we interfered with.

Importantly, based on c-Fos expression patterns and the specified coordinates, our study clearly avoids the rostralmost part of PVT. This can be clearly seen in our analysis of the regions activated by the POSE or inhibited by SwiChR (Supplemental Figure 2C). To explicitly address this, we have added the following sentence to the first paragraph of the Results section:

"Our manipulations and recordings were centered on middle portion of PVT (AP, BR: -0.8mm – 1.8mm) and did not include the rostral 500 µm sector of the nucleus or its most caudal end (see Methods section for details)"

C) Lack of controls: Several control experiments are necessary to support the assertion that post-stress event inhibition of PVT cells is critical. These include

1) assessing the effects of photoinhibition without POSE to show short/long-term effect of PVT inhibition itself,

2) determining the timing of inhibition by inhibition in pre- and during POSE, and

3) conducting long-term observations with YFP-infected animals (for Fig 8) to assess natural recovery from POSE stress.

We thank you for the suggestions to perform these control experiments. Please find our response below in this order.

1) Photoinhibition without POSE: To investigate the effects of PVT inhibition independent of the predator odor exposure, we conducted control experiments in SwiChR-injected animals exposed to a novel environment without predator odor (NOE group). Our novel environment control groups did not differ from the home cage control group in hyperventilation, sleep onset, c-Fos activity or CORT level (Fig. 1 G-M). Furthermore, as we show now in the revision (See New Supplementary Figure 9) they do not display long term changes in behavior. Following the exposure, we inhibited their PVT and observed the effects on behavior for 5 more days in the NOE SwiChR group. This manipulation resulted in decreased time to fall asleep (nest time), increased nest-building activity, and reduced freezing behavior (See New Supplementary Figure 9). Of these behaviors, freezing and nest-building showed significant changes. These findings indicate that a single event of PVT inhibition, even in the absence of a stressor (such as predator odor), can influence specific behaviors for a prolonged time and reduce the baseline level of stress related behaviors. These data confirmed our original conclusions on the prolonged effect of altering PVT activity, so thank you for this suggestion. We present the new findings on page 12, lines 271-278.

2) Timing of inhibition (Pre- and during POSE): The timing of inhibition is indeed crucial for understanding the role of the PVT in stress responses. As we describe in the Introduction we intentionally avoided inhibition of PVT/CR+ cells during the POSE. Since PVT/CR+ cells are known to be involved in stress, attention and detecting salience, reducing their activity during the stress exposure would reduce the effect of stress on the animals as well. As a consequence, any effect on the behavior is uninterpretable. In this condition alteration of behavior after stress can be caused both by the reduced impact of stress on the animal and the reduced effect of PVT/CR+ neurons on stress induced behavior. Applying photoinhibition after stress exposure clearly eliminates the first option, this is why we chose this approach. In fact, almost all previous studies on PVT and stress used the “inhibition-during-stress” protocol making their conclusions difficult to evaluate. Pre-stress inhibition (before POSE) presents an interesting but different concept, potentially leading to distinct outcomes. While this approach is beyond the scope of the present study, it represents a valuable direction for future research that we will consider exploring.

3) Long-term observations with EYFP mice: We conducted a follow-up experiment to assess natural recovery in EYFP animals 14 days after POSE. Contrary to expectations, our findings indicated that these stressed animals did not show significant recovery after 14 days compared to their post-POSE period. Specifically, the time spent with freezing and nest time were significantly elevated after 14 days of stress exposure compared to baseline conditions. This lack of recovery in EYFP mice contrasts with what might be expected in natural recovery scenarios, underscoring the prolonged impact of POSE and further validating the necessity and specificity of PVT inhibition in stress modulation. For reviewing purposes, we provide these data here.

**Fig. 2 for reviewing purposes only.** Long-term effect of POSE: Significant differences in nest time (A) and nest-building time (B) persist 12 days after exposure to the stressor. Freezing time (C) still shows a tendency to increase, but this does not reach significance.

Minor points:
a) Inconsistent figure labeling: There is inconsistency between figure panel labeling and the main text, with small case letters used in figure panels and large case letters in the legend.

Thank you for your observation regarding the inconsistency in figure labeling. We have corrected this by standardizing the labeling in the figure panels to match the uppercase format used in the legends.
b) Figure 1: There are two panel Ls, and panel M is missing.

Thank you for pointing out the error in Figure 1. We have corrected it by removing the duplicate panel L and adding the missing panel M.
c) Clarity on experimental timeline: From Figure 3 onwards, it is unclear what "Pre" and "Post" represent in each experiment. Specifying whether these refer to averages or results from specific days would enhance clarity.

Thank you for your feedback regarding the clarity of the experimental timeline in Figure 3. In the legend, we have indicated that "PRE" and "POST" values represent the averages of daily data for the corresponding PRE and POST days.

d) "CR-" neurons in Supplementary Figure 4: Should these be referred to as "ChR2-"? Clarification on distinguishing CR+ neurons without AAV infection and CR- neurons is needed.

Thank you for your comment regarding the "CR-" neurons in Supplementary Figure 4. Due to technical limitations, we cannot definitively determine whether these neurons are without AAV infection (and thus not expressing ChR2) or if they are indeed CR- neurons. Our histological data indicate that 98% of neurons showing CR immunoreactivity also express the reporter protein EYFP, indicating that they express ChR2. However, under in vivo experimental conditions, it is not possible to distinguish with 100% certainty between CR+ neurons without AAV infection and CR- neurons. This is why we referred to this neuron as a "putative CR-" neuron in the figure legend.
e) Line 559: Clarify whether "Same as H" refers to "same as B."

Thank you for your observation regarding line 559. We have corrected this by clarifying that "Same as H" refers to "same as B."

**Reviewer #3:**

Dr Jasz and colleagues present an interesting set of experiments focused on the role of the PVT in translating transient, acute stress to sustained behavioral llterations. Building on their prior work, they show that a 10 minute exposure to novelty coupled with a predator odour changes both the firing rate of calretinin cells in the PVT and stress/defensive behaviors. The show altered activity of the CR/PVT cells, and an effect of silencing these cells post hoc on subsequent behaviors. They suggest that the CR/PVT neuronal ensembles engage in protracted increased firing which, in turn, influences the consequences of stress.
The paper present an interesting concept an a significant amount of work. However, several points deserve the authors' attention.

First, the analyses are complex ad confusing. The authors are requested to put side by side the pre- and poststress behaviors after eYFP or inhibitory CR/PVT stimulation, and provide a two way ANOVA to look explicitly at the effects of stress and of the optogenetic manipulation. Without this, one is left trying to piece out facts in figures 1 and 5, which do not enable direct comparisons. Not clear why the authos use ttests and one way ANOVA.

We appreciate the reviewer’s comments regarding the complexity of the analyses and the request for additional statistical comparisons. The reviewer raises an important point about the need for clarity in comparing pre- and post-stress behaviors under different experimental conditions.

In our original analyses, we employed t-tests and one-way ANOVA due to the within-subject design, where each subject served as its own control across different time intervals. This auto-control approach is widely used in behavioral neuroscience and pharmacology to account for individual variability, as it provides a more sensitive measure of treatment effects by reducing noise associated with inter-individual differences.

We provide here the following references that support the auto-control designs:

Tse et al. 2023 explored the distinct phases of memory formation—encoding, storage, consolidation, and retrieval—and emphasizes the significance of novelty in enhancing memory retention through a within-subject design using optogenetics (Tse et al., 2023).

Gutzeit et al., 2020 found that optogenetic reactivation of socially active neuronal ensembles in the infralimbic prefrontal cortex (IL-PFC) mimicked the fear-reducing effects of cagemate presence in both learned and innate fear contexts. Utilizing a within-subject design, the research demonstrated that male and female mice exhibited reduced freezing behavior when tested with and without the presence of a cage mate in anxiety-provoking situations (Gutzeit et al., 2020).

Thomas et al. 2022 found that optical stimulation of lateral hypothalamic (LH) orexin/dynorphin inputs in the ventral tegmental area (VTA) potentiated mesolimbic dopamine neurotransmission in the nucleus accumbens (NAc) core, leading to real-time and conditioned place preference, as well as increased food cue-directed orientation in a Pavlovian conditioning procedure. Utilizing a within-subject design, the research revealed that the rewarding effects of LH orexin/dynorphin stimulation in the VTA were predominantly driven by orexin rather than dynorphin (Thomas et al., 2022).

Blanchard and Blanchard 1972 highlighted the value of within-subject designs in studying stress and fear responses, where each animal serves as its own control across different conditions (Blanchard & Blanchard, 1972).

Blanchard and Blanchard 1989 elaborated on the use of within-subject designs in complex behavioral paradigms, this study underscores the advantages of controlling for individual variability (Blanchard & Blanchard, 1989).

Holland and Gallagher 2004 discussed the utility of within-subject designs in neurobiological studies of behavior, particularly in experiments involving manipulation of neural circuits and subsequent behavioral analysis (Holland & Gallagher, 2004).

Cohen & Zohar, 2004 studied supports the use of within-subject designs in stress research, particularly in models of post-traumatic stress disorder (PTSD), where behavioral baselines are crucial for assessing the impact of stressors (Cohen & Zohar, 2004).

In response to the reviewer’s request, we conducted a two-way ANOVA to directly compare pre- and post-stress behaviors and to account for both the effect of time and the optogenetic manipulation (Group Factor). Although this analysis is not typically applied in within-subject designs, we present it for the reviewer’s consideration.

For nest-building behavior, the analysis revealed that there was no significant main effect of time (F(1,26) = 1.375, p = 0.2517) or Group Factor (F(1,12) = 3.439, p = 0.0884). Additionally, there was no significant interaction between time and Group Factor (F(1,12) = 1.181, p = 0.2984). Post-hoc Fisher’s LSD analysis showed that the comparison between the EYFP and SwiChR groups was not significant pre-stress (p = 0.5972), but there was a trend toward significance post-stress (P = 0.0596).

For nest time, the two-way ANOVA indicated that the main effect of time was not significant (F(1,26) = 1.736, p = 0.1991), but there was a significant main effect of Group Factor (F(1,12) = 8.582, p = 0.0126). The interaction between time and Group Factor was not significant (F(1,12) = 1.200, p = 0.2948). Post-hoc analysis showed no significant difference between the EYFP and SwiChR groups pre-stress (P = 0.2190), but a significant difference was found post-stress (P = 0.0147).

For freezing time, we found significant main effects of both time (F(1,38) = 8.864, p = 0.0050) and Group Factor (F(1,38) = 11.67, p = 0.0015 P=0.0015). The interaction between time and Group Factor was also significant (F(1,38) = 13.32, p=0.0008). Post-hoc analysis revealed no significant difference between the EYFP and SwiChR groups pre-stress (p= 0.8700), but a highly significant difference post-stress (p < 0.0001).

These two-way ANOVA results emphasize the significant effects of optogenetic manipulation, particularly in freezing behavior, where both the main effects and the interaction were significant. While nest-building and nest time showed trends and significance primarily in the post-stress condition, this suggests that these behaviors are more sensitive to stress manipulations.

In conclusion, the use of t-tests and one-way ANOVA in our original analysis was consistent with the principles of within-subject designs in behavioral neuroscience. However, the two-way ANOVA results, provided for the reviewer’s clarity, offer further statistical insights and facilitate direct comparisons between PRE and POST conditions.

We should note that due to its inherent properties, two-way ANOVA may struggle to detect main effects if the data exhibit large variability between individual subjects. Two-way ANOVA can still detect significant effects if the variability is within the expected range for the given sample size and the effects are large enough. In our study, we employed a unique behavioral approach by measuring spontaneous behaviors in the home cage of animals over several days before and after stress. We believe this method provides the most relevant insight into stress response, particularly in stress research involving the PVT, where similar approaches are rare. Most studies assess stress in controlled, instrumental conditions outside the home cage (e.g., conditional fear boxes, elevated plus maze, light-dark box), which introduces additional stress and may not be as accurate as monitoring spontaneous behavior in the home cage.

In our experiments spontaneous behavior in mice displayed high variability. As shown on our graphs, tenfold differences between mice within the same cohort were not uncommon. Despite this variability, our within-subject control design clearly shows that EYFP-expressing animals displayed significantly worse outcomes across all measures after the stress, while SwiChR animals did not. This represents a robust and clear effect. However, due to the large inter-animal variability, the statistical power of the ANOVA may be limited in detecting such effects, even though the effect is visually apparent.

A second general question pertains to the relation of the current work with the Matyas 2018 paper. There, CR neurons in the dorsomedial thalamus are implicated in arousal. Interestingly, some demonstrate prolonged firing in response to a tail pinch. The term PVT is not used in the 2018 paper, though clearly, the CR/PVT neurons are involved. Might the authors clarify the relation of the two papers, and the novelty of the current work?
Note that the authors move mice from the home cage to a new one when presenting a predator odour. Thus, they generate arousal together with stress. In view of the role of anterior PVT in arousal, this is a confounder. While the authors show that the new cage by itself does not generate stress behaviors, they cannot claim that the novelty is unimportant. Indeed, they are measuring the combined effects of predator odour and novelty.

We appreciate the reviewer's insightful question regarding the relationship between our current study and the (Mátyás et al., 2018) paper. We would like to clarify the distinctions and novel contributions of our research in this context.

First, we clarify the different nomenclatures used in the two papers. In (Mátyás et al., 2018), we found that CR+ neurons in the PVT and in the intralaminar nuclei (centrolateral, centromedial and paracentral) have similar reactivity to day/light changes, to the increasing levels of arousing stimuli and display similar input selectivity. Moreover, exactly the same input selectivity was present in the centrolateral and PVT of the human material as well. This prompted us to propose in the Discussion of (Mátyás et al., 2018), that “…CR content rather than location of these cells in a specific thalamic nucleus is a key trait for anatomically defining this system”. In other words, a CR+ neuron in the PVT and intralaminar nucleus will have more similar functions than a CR+ and a CR- cell in the same nucleus. This idea is reminiscent of the original “matrix and core” distinction based on calbindin and parvalbumin content as proposed by the late Ted Jones (Jones, 2001). The different nomenclature (PVT) we used now can be explained by two facts. First, in this new paper we confined all the recording, measurements and interaction to PVT thus DMT would have been an inappropriate term here. Second, the “DMT” nomenclature has not been widely accepted since the publication of (Mátyás et al., 2018).

Concerning tail-pinch. We are glad that our reviewer read our previous paper in detail. Indeed, in Suppl Fig 3 of (Mátyás et al., 2018) we demonstrated prolonged increase in the firing rate of some CR+ neurons following a 30-second tail pinch in anesthetized animals. First, we need to emphasize that this effect never lasted longer than 1 minute. In contrast, in our present paper we found elevated firing rates for up to 5 days after the stress insult in freely moving animals. Second, in Matyas et al., the elevated firing rate was tightly linked to a decreased delta activity in the cortex. Thus, it might have been maintained by the aroused cortex. In contrast, in the present study, in the comparable naturally sleeping state, even though PVT/CR+ cells fired more (Figure 4) the depth of sleep did not change (Figure 3J).

Finally, the insults (tail pinch in anesthesia vs fox odor exposure in awake animals) are not comparable. In any case, the data of (Mátyás et al., 2018) indicate that these cells might have a tendency for a prolonged response to an insult, but the two papers work in completely different regimes. Our present study directly examines the role of PVT/CR+ neurons under conditions of POSE. This model of stress, involving a 10-minute exposure to predator odor, is significantly more prolonged and aversive than the brief tail pinch used in Matyas et al. Our findings provide novel insights into how PVT/CR+ contribute to both arousal and stress responses in a naturalistic threat context. The prolonged nature of POSE and its robust activation of stress responses at the hormonal, neuronal, and behavioral levels differentiate it substantially from the momentary arousal induced by tail pinch. Therefore, these stimuli engage different aspects of the stress and arousal spectrum.

Furthermore, the present study uniquely assesses the temporal dynamics of stress responses induced by POSE, examining changes over several days. This long-term analysis contrasts with the immediate and short-lived responses observed in the Matyas study and adds another layer of novelty to our work. By tracking the persistence of stress-related behaviors and neural activity, we provide a more comprehensive understanding of how PVT/CR+ neurons are involved in sustained stress responses, something that has not been explored in the context of tail pinch-induced arousal.

We acknowledge the reviewer's concern regarding the potential confounding effect of environmental novelty when moving mice from their home cage to a new one during POSE. While novelty can indeed contribute to arousal, we have taken rigorous steps to check for this confounder. We conducted experiments with a novelty control group (NOE), where mice were exposed to the same new environment without the predator odor, expressing EYFP for comparison. Our results showed no significant changes in any behavioral variables in the novelty control group, indicating that the observed effects in the POSE group are not merely to environmental novelty (Supplementary Figure 6). Nevertheless, in addition to fox odor and novel cage, high-intensity light and the sound of the fume hood were also present during the stress exposure. These served to create a highly controlled and robust stress environment. We consider that the long-term effects of stress exposure are due to the combined effect of the fox odor and the new environment.

Since there is no response to stress without increased arousal, strong distinction of these two processes under these conditions may be confounding. Indeed, we think that PVT/CR+ cells participate in both processes, (Mátyás et al., 2018), demonstrate their involvement in normal arousal, while Jasz and Biro et al., demonstrate their involvement in stress-induced alteration of behavior.

In summary, while (Mátyás et al., 2018) provided important insights into the role of CR neurons in arousal within the dorsomedial thalamus, our study significantly extends this work by focusing on the PVT and examining stress responses to a naturalistic and prolonged threat. The differences in the nature and duration of the stressors, as well as our exploration of long-term behavioral and neuronal changes, underscore the novelty and significance of our findings. We believe that our work offers a unique and scientifically rigorous contribution to understanding the role of PVT neurons in stress and arousal.

They choose a single type of arousal/ stress, is this generalizable to others?

We appreciate the reviewer's inquiry regarding the generalizability of our findings to other types of arousal or stress. While our study focused on a single stressor—predator odor exposure (POSE)—we believe our results are generalizable to other forms of acute stress based on earlier studies that have already examined the role of PVT in a wide range of stressors. For example, in Zhao et al. (J. Zhao et al., 2022), chemogenetic inhibition of CeA-projecting PVT neurons alleviated stress responses across different acute stress models, including cage change, acute restraint, and exposure to TMT (a predator odor). Similarly, Zhao et al. (Zhao et al., 2021) examined chronic unpredictable stress (CUS) and found a significant increase in ΔFosB expression, a marker of sustained neural activation, in all subregions of the PVT 24 hours after CUS. Finally in Zhao et al. (D. Zhao et al., 2022), acute restraint stress led to a marked increase in c-Fos-positive neurons within different PVT subregions. Our own previous (Mátyás et al., 2018) study demonstrated that c-Fos expression in PVT correlated with the intensity of stress (home cage, handling, foot shock) In conclusion, based on the existing literature and the PVT’s known role in stress regulation across various conditions, we believe our results are indeed generalizable to other stressors. While further research using a variety of stress models would be valuable, the consistency of PVT involvement across different stress paradigms, combined with the unique temporal dynamics captured in our study, strongly suggests that the findings from our POSE model are relevant to a wider range of stress conditions. As a final note, our study is unique in analyzing short- and long-term changes in spontaneous behavior and PVT firing activity in freely moving animals together with alterations in c-Fos activity, hormone levels, receptor expression and depth of sleep in experimental and several control cohorts. Replication of the entire protocol for another stressor is not feasible and beyond the scope of the present account.

Both Bhatnagar and Do Monte suggest posterior PVT is involved in stress encoding. Where in PVT did the authors implant their hardware? They provide coordinates, but in view of the striking divergence of different atlases for delineating the PVT, can the authors specify what part of that elongated structure they tackled?

We thank the reviewer for their thoughtful question regarding the regional specificity within the PVT, particularly in light of the work by Bhatnagar and Do Monte, which highlights the role of the posterior PVT (pPVT) in stress encoding. We appreciate the opportunity to clarify the specific regions of the PVT that were targeted in our study.

In our study, we meticulously detailed the coordinates for all manipulations, ensuring transparency about the specific areas of the PVT that were involved. We recognize that the border between the anterior PVT (aPVT) and pPVT can vary between different atlases, and this has been a point of significant debate in the field. However, based on our coordinates and c-Fos expression patterns, our primary focus was, what is generally called the pPVT. The aPVT, particularly its rostralmost part, which receives selective subicular input (Canteras & Swanson, 1992), was not included in this study.

We further support this regional specificity through various methodological details and supplementary figures. For instance, in the behavioral, corticosterone, c-Fos, and EEG experiments, optic fibers were implanted at specific coordinates (AP: -0.9 and -1.4, ML: 0, DV: 2.8, 10° angle) to target the PVT. Tetrodes in electrophysiological recordings were also implanted at similar coordinates (AP: -0.9, ML: 0, DV: 2.7, 9.5° angle) and gradually adjusted until the start of the experiment. We provide histological evidence of the implantation tracks in several figures, including Figures 1, 7, and Supplementary Figures 2, 3, 6, 9, 11, and 12. These figures confirm that our manipulations were predominantly within the pPVT, aligning with the focus of our study.

To further strengthen our manuscript, we will explicitly state in the revised version that (page 5, lines 108-110):

“Our manipulations and recordings were centered on middle portion of PVT (AP, BR: -0.8mm – 1.8mm) and did not include the rostral 500 µm sector of the nucleus or its most caudal end (see Methods section for details).”

This clarification will help readers understand the specific regional focus of our study, particularly in relation to the involvement of the pPVT in stress responses. We believe that these clarifications, along with the additional details provided, will address the reviewer's concerns and underscore the regional specificity of our study within the PVT. Please also refer to our response on the same topic above (Reviewer 2 Question B).

While the authors identify an interesting potential mechanism for a more enduring effect of PVT on stress, they seem to be unaware of recent work using genetic tagging, which shows that PVT encodes transient stress early-in life, and translates it to enduring changes in adult behavior. This work should be acknowledged.

Thank you for highlighting the recent work by Kooiker et al., which provides valuable insights into the PVT's role in encoding early-life adversity (ELA) and its long-term impact on adult behavior (Kooiker et al., 2024). We have incorporated this important study into our discussion (page 19, lines 458-460), as it aligns with our findings on the PVT's involvement in stress responses.

In their study, "Paraventricular Thalamus Neuronal Ensembles Encode Early-life Adversity and Mediate the Consequent Sex-dependent Disruptions of Adult Reward Behaviors" Kooiker et al. demonstrated that the PVT is selectively activated by early life adversities (ELA). While the conclusions of Kookier et al are certainly not against our present data we should be aware of the differences between these approaches. Mice are exposed to ELA during several days during development. In contrast, we expose adult animals to an innate stressor for 10 minutes. Even if in both cases long term changes are recorded in PVT the underlying mechanisms and the impact on the forebrain network can be quite different.

In view of the complexity and heterogeneity of the PVT, the authors are requested to provide actual photomicrographs of their targeting, rather than schematics.

Thank you for your suggestion regarding the inclusion of photomicrographs to demonstrate the precision of our targeting within the PVT. We understand the importance of accurately representing the complexity and heterogeneity of this region.

In response, we have provided actual photomicrographs in the revised manuscript, specifically in Figures 1 and 7, as well as Supplementary Figure 2, 6, 9. These images clearly illustrate the targeting of our manipulations within the PVT, offering a detailed view of the specific regions where our hardware, such as optic fibers and tetrodes, was implanted.

We believe that we have provided an unusually detailed demonstration of both photomicrographs and diagrams. The localization of fiber optics and other devices is depicted in Figures 1, 7, and Supplementary Figures 2, 3, 6, 9, 11, and 12, which goes far beyond the average level of detail typically provided. In addition, as detailed in our response to Review 2/point A we performed an analysis of c-Fos expression to measure the spatial extent of our SwiChR inhibition. These additions enhance the transparency and robustness of our findings, effectively addressing concerns related to the regional specificity of our interventions.

While the authors ascribe important functions for the augmented activity in PVT CR cells do they know what instigates it? What stops it? A speculation in the discussion would be helpful. The changes in GABAA alpha 1 and gamma 2 by themselves do not answer these questions

Thank you for your insightful question regarding the factors that instigate and terminate the augmented activity in PVT CR cells. We agree that understanding the initiation and cessation mechanisms of this activity is crucial for a comprehensive understanding of PVT's role in stress responses.

In our study, we observed that predator odor exposure (POSE) triggers the activation of CR cells in the PVT, suggesting that this stressor acts as a primary instigator. This aligns with the PVT's established role in processing emotionally salient stimuli, particularly those linked to fear and arousal. Although the precise upstream mechanisms initiating this activity remain to be fully elucidated, it is likely that the activation results from a combination of sensory inputs and neuromodulatory influences from regions such as the amygdala or hypothalamus.

Regarding the cessation of this augmented activity, it is plausible that both intrinsic and extrinsic factors are involved. Intrinsic factors might include the downregulation of excitatory neurotransmission or the activation of inhibitory circuits within the PVT. Extrinsic factors could involve feedback mechanisms from other brain regions, such as the prefrontal cortex or hippocampus, which exert top-down control over stress responses.

While changes in GABA A receptor subunits, specifically α1 and γ2, suggest a shift in inhibitory tone within the PVT, these alterations alone do not fully explain the dynamics of CR cell activity. We speculate that the termination of this activity might involve the restoration of homeostatic balance through inhibitory feedback loops, potentially mediated by increased GABAergic transmission or other neuromodulatory systems that counteract the heightened arousal state.

We have incorporated a discussion of these speculative mechanisms on pages 20-21, lines 486-489, of our manuscript. Please also see the text on pages 19 to 20, lines 461-470. This paragraph aims to provide a broader context for understanding the initiation and cessation of CR cell activity in the PVT, acknowledging the complexity of these processes and suggesting potential avenues for further research. We hope this addition clarifies our approach and addresses your query effectively.

**Reviewer** 4:
The topic of this manuscript seems interesting. But, I found a few potential major issues:

* The stress paradigm: The “stress” is exposure to a predator odor. There are several articles (e.g., Staples, 2008; McGregor, 2002; Hacquemand, 2010) suggesting that fox odor (in contrast to cat saliva or other cat odors) is not really a fear-inducing stressor but just a bad smelling odor, especially at high concentration (youused 100%). The 2-Methyl-2-thiazoline, which is used instead of the commonly-studied fox odor chemical TMT (2,4,5-Trimethylthiazole), has been shown to produce strong freezing responses (Isosaka, 2015 PMID: 26590419). Is this odor, that is not a component of fox urine, acting as a bad smell rather than an innate fear signal?

Thank you for your insightful comment regarding the use of predator odor in our stress paradigm. We appreciate your attention to the validity of using 2-Methyl-2-thiazoline (2-MT) as a stressor. 2-MT is a predator odor related kairomone-derived compound and as such evokes the same innate fear response. Some studies have questioned the effectiveness of fox odor as a fear-inducing stressor compared to other predator-related cues. Our decision to use 2-MT was based on its well-documented and has been shown to induce dose-dependent fear and stress responses, including freezing, avoidance of the odor source, hypothermia, hypometabolism, and increases in corticosterone (Endres et al., 2005; Isosaka et al., 2015; Matsuo et al., 2021; Rosen et al., 2015; Takahashi et al., 2005; Wallace & Rosen, 2000; Wang et al., 2018). Unlike fox odor, which may have variable effectiveness in different contexts, 2-MT is known to reliably induce robust stress responses, including significant behavioral and physiological changes.

For example, research by Isosaka et al. (2015) demonstrated that 2-MT elicits pronounced freezing responses in rodents, establishing its effectiveness as a potent stressor (Isosaka et al., 2015). In this paper both substances have been used to induce stress response. Our own data supports the effectivity of 2MT. We observed substantial increases in corticosterone levels and significant changes in stress-related behaviors following 2-MT exposure. These findings confirm that 2-MT is functioning as an effective stressor rather than just a noxious odor. We hope this clarification addresses your concerns and reinforces the validity of our chosen stress paradigm. We cite the relevant papers on 2-MT in the text on page 5, lines 111-112.

Figure 1 controls: You compare post-stress behavior (hyperventilation and sleep onset) in EYFP to SwiChR mice, but do not have unstressed mice to compare to. Comparing pre to post stress is problematic for many of these analyses, because animals also change over time and with repeated handling and testing. Please compared to unstressed controls for many of these analyses.

We appreciate the importance of comparing post-stress behavior not only to pre-stress baselines but also to unstressed controls to account for potential changes due to repeated handling and testing.

To address this concern, we conducted additional analyses using a novel environment control group during the post-stress 1-hour period. Our findings indicate that exposure to a novel environment did not produce significant changes in the measured variables and did not differ from the SwiChR-inhibited group (Figure 1G and H). This suggests that the observed effects in the experimental groups are indeed attributable to the stressor and not to the novelty or repeated handling.

We have incorporated these findings into the revised manuscript to strengthen the validity of our conclusions. Specifically, we included a comparison with unstressed controls in the relevant analyses to ensure a robust interpretation of the data. This additional control analysis confirms that the changes observed post-stress are stress-specific and not artifacts of the experimental procedure. The data are shown in Figures 1G and H and are described in the text on page 6, lines 132-136.

Figure 5 compared to Figure 3: You test SwiChR mice pre and post stress in Figure 5, but do not compare these results directly to the EYFP control animals shown in Figure 3. A change in one group and lack of change in another does not demonstrate a difference between groups. Please directly compare these groups statistically.

Thank you for your observation regarding the need for a direct comparison between the SwiChR mice in Figure 5 and the EYFP control animals in Figure 3. As mentioned in our response to the first question of Reviewer 3 our initial analyses were based on a within-subject design, which compared pre- and post-stress behaviors within each group. This approach allowed us to control for individual variability and focus on time-dependent effects.

However, in response to your request, we performed a two-way ANOVA to directly compare the SwiChR and EYFP groups, considering both the effects of time (pre- and post-stress) and group (SwiChR vs. EYFP). This analysis showed significant differences between the groups, particularly in freezing behavior, where we found a significant interaction between time and group (as detailed in the previous answer). These results provide a clearer statistical comparison between the two groups, addressing your concern about demonstrating differences between the groups.

In conclusion, the two-way ANOVA now included in the analysis allows for direct comparisons between the SwiChR and EYFP groups, as per your suggestion.

References

Blanchard, D. C., & Blanchard, R. J. (1972). Innate and conditioned reactions to threat in rats with amygdaloid lesions. *J Comp Physiol Psychol*, *81*(2), 281-290. <https://doi.org/10.1037/h0033521>

Blanchard, D. C., Griebel, G., & Blanchard, R. J. (2001). Mouse defensive behaviors: pharmacological and behavioral assays for anxiety and panic. *Neurosci Biobehav Rev*, *25*(3), 205-218. <https://doi.org/10.1016/s0149-7634(01)00009-4>

Blanchard, R. J., & Blanchard, D. C. (1989). Antipredator defensive behaviors in a visible burrow system. *J Comp Psychol*, *103*(1), 70-82. <https://doi.org/10.1037/0735-7036.103.1.70>

Canteras, N. S., & Swanson, L. W. (1992). Projections of the ventral subiculum to the amygdala, septum, and hypothalamus: a PHAL anterograde tract-tracing study in the rat. *J Comp Neurol*, *324*(2), 180-194. <https://doi.org/10.1002/cne.903240204>

Cohen, H., & Zohar, J. (2004). An animal model of posttraumatic stress disorder: the use of cut-off behavioral criteria. *Ann N Y Acad Sci*, *1032*, 167-178. <https://doi.org/10.1196/annals.1314.014>

Endres, T., Apfelbach, R., & Fendt, M. (2005). Behavioral changes induced in rats by exposure to trimethylthiazoline, a component of fox odor. *Behavioral Neuroscience*, *119*(4), 1004-1010. <https://doi.org/10.1037/0735-7044.119.4.1004>

Gutzeit, V. A., Ahuna, K., Santos, T. L., Cunningham, A. M., Sadsad Rooney, M., Muñoz Zamora, A., Denny, C. A., & Donaldson, Z. R. (2020). Optogenetic reactivation of prefrontal social neural ensembles mimics social buffering of fear. *Neuropsychopharmacology*, *45*(6), 1068-1077. <https://doi.org/10.1038/s41386-020-0631-1>

Holland, P. C., & Gallagher, M. (2004). Amygdala-frontal interactions and reward expectancy. *Curr Opin Neurobiol*, *14*(2), 148-155. <https://doi.org/10.1016/j.conb.2004.03.007>

Hua, R., Wang, X., Chen, X., Wang, X., Huang, P., Li, P., Mei, W., & Li, H. (2018). Calretinin Neurons in the Midline Thalamus Modulate Starvation-Induced Arousal. *Curr Biol*, *28*(24), 3948-3959.e3944. <https://doi.org/10.1016/j.cub.2018.11.020>

Isosaka, T., Matsuo, T., Yamaguchi, T., Funabiki, K., Nakanishi, S., Kobayakawa, R., & Kobayakawa, K. (2015). Htr2a-Expressing Cells in the Central Amygdala Control the Hierarchy between Innate and Learned Fear. *Cell*, *163*(5), 1153-1164. <https://doi.org/10.1016/j.cell.2015.10.047>

Jones, E. G. (2001). The thalamic matrix and thalamocortical synchrony. *Trends Neurosci*, *24*(10), 595-601. <https://doi.org/10.1016/s0166-2236(00)01922-6>

Kooiker, C. L., Birnie, M. T., Floriou-Servou, A., Ding, Q., Thiagarajan, N., Hardy, M., & Baram, T. Z. (2024). Paraventricular Thalamus Neuronal Ensembles Encode Early-life Adversity and Mediate the Consequent Sex-dependent Disruptions of Adult Reward Behaviors. *bioRxiv*. <https://doi.org/10.1101/2024.04.28.591547>

Kvitsiani, D., Ranade, S., Hangya, B., Taniguchi, H., Huang, J. Z., & Kepecs, A. (2013). Distinct behavioural and network correlates of two interneuron types in prefrontal cortex. *Nature*, *498*(7454), 363-366. <https://doi.org/10.1038/nature12176>

Matsuo, T., Isosaka, T., Tang, L., Soga, T., Kobayakawa, R., & Kobayakawa, K. (2021). Artificial hibernation/life-protective state induced by thiazoline-related innate fear odors. *Commun Biol*, *4*(1), 101. <https://doi.org/10.1038/s42003-020-01629-2>

Mátyás, F., Komlósi, G., Babiczky, Á., Kocsis, K., Barthó, P., Barsy, B., Dávid, C., Kanti, V., Porrero, C., Magyar, A., Szűcs, I., Clasca, F., & Acsády, L. (2018). A highly collateralized thalamic cell type with arousal-predicting activity serves as a key hub for graded state transitions in the forebrain. *Nature Neuroscience*, *21*(11), 1551-1562. <https://doi.org/10.1038/s41593-018-0251-9>

Rosen, J. B., Asok, A., & Chakraborty, T. (2015). The smell of fear: innate threat of 2,5-dihydro-2,4,5-trimethylthiazoline, a single molecule component of a predator odor. *Front Neurosci*, *9*, 292. <https://doi.org/10.3389/fnins.2015.00292>

Takahashi, L. K., Nakashima, B. R., Hong, H., & Watanabe, K. (2005). The smell of danger: a behavioral and neural analysis of predator odor-induced fear. *Neurosci Biobehav Rev*, *29*(8), 1157-1167. <https://doi.org/10.1016/j.neubiorev.2005.04.008>

Thomas, C. S., Mohammadkhani, A., Rana, M., Qiao, M., Baimel, C., & Borgland, S. L. (2022). Optogenetic stimulation of lateral hypothalamic orexin/dynorphin inputs in the ventral tegmental area potentiates mesolimbic dopamine neurotransmission and promotes reward-seeking behaviours. *Neuropsychopharmacology*, *47*(3), 728-740. <https://doi.org/10.1038/s41386-021-01196-y>

Tse, D., Privitera, L., Norton, A. C., Gobbo, F., Spooner, P., Takeuchi, T., Martin, S. J., & Morris, R. G. M. (2023). Cell-type-specific optogenetic stimulation of the locus coeruleus induces slow-onset potentiation and enhances everyday memory in rats. *Proc Natl Acad Sci U S A*, *120*(46), e2307275120. <https://doi.org/10.1073/pnas.2307275120>

Wallace, K. J., & Rosen, J. B. (2000). Predator odor as an unconditioned fear stimulus in rats: elicitation of freezing by trimethylthiazoline, a component of fox feces. *Behavioral Neuroscience*, *114*(5), 912-922. <https://doi.org/10.1037//0735-7044.114.5.912>

Wang, Y., Cao, L., Lee, C. Y., Matsuo, T., Wu, K., Asher, G., Tang, L., Saitoh, T., Russell, J., Klewe-Nebenius, D., Wang, L., Soya, S., Hasegawa, E., Chérasse, Y., Zhou, J., Li, Y., Wang, T., Zhan, X., Miyoshi, C., . . . Liu, Q. (2018). Large-scale forward genetics screening identifies Trpa1 as a chemosensor for predator odor-evoked innate fear behaviors. *Nature Communications*, *9*(1), 2041. <https://doi.org/10.1038/s41467-018-04324-3>

Zhao, D., Liu, C., Cui, M., Liu, J., Meng, F., Lian, H., Wang, D., Hu, F., Liu, D., & Li, C. (2021). The paraventricular thalamus input to central amygdala controls depression-related behaviors. *Exp Neurol*, *342*, 113744. <https://doi.org/10.1016/j.expneurol.2021.113744>

Zhao, D., Wang, D., Wang, W., Dai, J., Cui, M., Wu, M., Liu, C., Liu, J., Meng, F., Wang, K., Hu, F., Liu, D., Qiu, C., Li, W., & Li, C. (2022). The altered sensitivity of acute stress induced anxiety-related behaviors by modulating insular cortex-paraventricular thalamus-bed nucleus of the stria terminalis neural circuit. *Neurobiol Dis*, *174*, 105890. <https://doi.org/10.1016/j.nbd.2022.105890>

Zhao, J., Liu, C., Zhang, F., Zheng, Z., Luo, F., Xia, J., Wang, Y., Zhang, Z., Tang, J., Song, Z., Li, S., Xu, K., Chen, M., Jiang, C., He, C., Tang, L., Hu, Z., Gao, D., & Ren, S. (2022). A paraventricular thalamus to central amygdala neural circuit modulates acute stress-induced heightened wakefulness. *Cell Rep*, *41*(11), 111824. <https://doi.org/10.1016/j.celrep.2022.111824>
